# Mechanisms Underlying Synergistic Killing of Polymyxin B in Combination with Cannabidiol against *Acinetobacter baumannii*: A Metabolomic Study

**DOI:** 10.3390/pharmaceutics14040786

**Published:** 2022-04-03

**Authors:** Maytham Hussein, Rafah Allobawi, Irini Levou, Mark A. T. Blaskovich, Gauri G. Rao, Jian Li, Tony Velkov

**Affiliations:** 1 Department of Biochemistry and Pharmacology, School of Biomedical Sciences, Faculty of Medicine, Dentistry and Health Sciences, The University of Melbourne, Parkville, VIC 3010, Australia; maytham.hussein@unimelb.edu.au (M.H.); rafah.allobawi@unimelb.edu.au (R.A.); ilevou@student.unimelb.edu.au (I.L.); 2 Institute for Molecular Bioscience, The University of Queensland, Brisbane, QLD 4072, Australia; m.blaskovich@imb.uq.edu.au; 3 Division of Pharmacotherapy and Experimental Therapeutics, Eshelman School of Pharmacy, University of North Carolina, Chapel Hill, NC 27599, USA; gaurirao@live.unc.edu; 4 Monash Biomedicine Discovery Institute, Department of Microbiology, Monash University, Clayton, VIC 3800, Australia

**Keywords:** antimicrobial resistance, antimicrobial peptides, cannabidiol, metabolomics, MDR Gram-negative

## Abstract

Polymyxins have resurged as the last-resort antibiotics against multidrug-resistant *Acinetobacter baumannii*. As reports of polymyxin resistance in *A. baumannii* with monotherapy have become increasingly common, combination therapy is usually the only remaining treatment option. A novel and effective strategy is to combine polymyxins with non-antibiotic drugs. This study aimed to investigate, using untargeted metabolomics, the mechanisms of antibacterial killing synergy of the combination of polymyxin B with a synthetic cannabidiol against *A. baumannii* ATCC 19606. The antibacterial synergy of the combination against a panel of Gram-negative pathogens (*Acinetobacter baumannii*, *Klebsiella pneumoniae* and *Pseudomonas aeruginosa*) was also explored using checkerboard and static time-kill assays. The polymyxin B–cannabidiol combination showed synergistic antibacterial activity in checkerboard and static time-kill assays against both polymyxin-susceptible and polymyxin-resistant isolates. The metabolomics study at 1 h demonstrated that polymyxin B monotherapy and the combination (to the greatest extent) significantly perturbed the complex interrelated metabolic pathways involved in the bacterial cell envelope biogenesis (amino sugar and nucleotide sugar metabolism, peptidoglycan, and lipopolysaccharide (LPS) biosynthesis), nucleotides (purine and pyrimidine metabolism) and peptide metabolism; notably, these pathways are key regulators of bacterial DNA and RNA biosynthesis. Intriguingly, the combination caused a major perturbation in bacterial membrane lipids (glycerophospholipids and fatty acids) compared to very minimal changes induced by monotherapies. At 4 h, polymyxin B–cannabidiol induced more pronounced effects on the abovementioned pathways compared to the minimal impact of monotherapies. This metabolomics study for the first time showed that in disorganization of the bacterial envelope formation, the DNA and RNA biosynthetic pathways were the most likely molecular mechanisms for the synergy of the combination. The study suggests the possibility of cannabidiol repositioning, in combination with polymyxins, for treatment of MDR polymyxin-resistant Gram-negative infections.

## 1. Introduction

Antimicrobial resistance has become a major threat to health and economic wellbeing worldwide [1]. The rapid emergence of MDR Gram-negative pathogens (e.g., carbapenem-resistant *Acinetobacter baumannii*) that cause difficult or impossible-to-treat infections is gaining momentum [1,2]. *A. baumannii* is often associated with serious nosocomial and community-acquired infections e.g., pneumonia and blood stream infections [3,4]. In the 1970s, this notorious pathogen was thought to be sensitive to most antibiotics, but today it appears to have extensive resistance to most currently used antibiotics, including polymyxins [5]. The World Health Organization (WHO) has, therefore, listed this pathogen as one of the top-priority pathogens that urgently need new antibiotics [6]. The clinical pipeline for new antimicrobials is dry; therefore, the development of novel therapeutic strategies to combat these deadly pathogens is warranted [1,7].

Colistin, also known as polymyxin E, and polymyxin B (cyclic lipopeptides) are last-resort antibiotics for Gram-negative pathogens resistant to essentially all other antibiotics [8]. It is purported that polymyxins exert their antimicrobial action primarily by disorganizing the Gram-negative outer membrane (OM) via direct interaction with the lipopolysaccharide (LPS); however, the precise mechanism of action is still uncertain [9,10]. Worryingly, there has been an increase in reports of polymyxin resistance among Gram-negative bacteria, including *A. baumannii*, particularly following polymyxin monotherapy [11,12,13]. The complete LPS loss or lipid A modification with positively charged moieties (phosphoethanolamine (PEtN) or galactosamine (GalN)) are considered the most common modes of polymyxin resistance observed in *A. baumannii* [12,14,15].

A cost-effective strategy that is gaining momentum is the combination of antibiotics with non-antibiotic compounds that facilitate the exertion of ‘off-target’ antibacterial properties against the bacterial cell [16,17,18,19,20,21,22,23]. The advent of metabolomics, comprising cutting-edge bioanalytical techniques and bioinformatics, provides a new strategy for fathoming the complex interactions between cellular processes and elucidating the complex modes of action following antibiotic exposure [24,25,26,27].

*Cannabis sativa* is an herbaceous plant that has been used as a medicinal and recreational agent for centuries worldwide [28]. This complex plant comprises more than 400 chemical entities, including many terpenes and approximately 180 cannabinoids (e.g., Δ9 tetrahydrocannabinol (Δ9 THC) and cannabidiol (CBD)) [29,30]. The antibacterial activity of cannabinoids was reported in detail for the first time in the 1950s [31,32]. Later, in 1976, van Klingeren et al. reported that ∆9-THC and CBD displayed bacteriostatic and bactericidal effects against a panel of Gram-positive pathogens such as staphylococci and streptococci [33]. However, the cannabinoids’ antimicrobial effects had been largely overlooked until 2008, when the antibacterial activity of five major cannabinoids (CBD, THC, cannabichromene (CBC), cannabigerol (CBG), and cannabinol (CBN)) were tested against a wide range of methicillin-resistant *Staphylococcus aureus* (MRSA) strains. The study revealed that all compounds possessed low MIC values (0.5–2 μg/ mL) [34]. As a result, there has been growing interest in the antibacterial properties of cannabinoids in the quest to discover new lead compounds. Subsequent and more recent studies have provided further insights into the chemical and antimicrobial characterization of various chemical entities in *Cannabis sativa* as well as synthetic CBD [35,36,37].

Mark and co-workers recently confirmed their previous findings by testing the killing activity of CBD against highly resistant *Staphylococcus aureus* (e.g., methicillin-resistant *Staphylococcus aureus* MRSA), *Streptococcus pneumoniae*, and *Clostridioides difficile* as well as the ‘urgent threat’ Gram-negative pathogen *Neisseria gonorrhoeae*. They also noticed that cannabinoids were able to effectively disorganize biofilms and sustain their antibacterial effects under extended exposure conditions [38]. Furthermore, in line with previous reports, the study showed that CBD was inactive against the most notorious Gram-negative pathogens, namely *K. pneumoniae*, *P. aeruginosa* and *A. baumannii*. However, combining CBD with colistin and polymyxin B caused a synergistic killing effect against *A. baumannii* and *K. pneumoniae,* suggesting that polymyxins might permeabilize the outer membrane and enable the entry of CBD to exert its action [38,39]. The mechanistic studies (including radiolabeled macromolecular synthesis assays, membrane depolarization assays and fluorescent microscopy) of CBD against *S. aureus* indicated that CBD disrupts the bacterial cytoplasmic membrane, though the precise mode of action is still elusive and further studies at the molecular level are warranted [38].

The aim of the present study was to evaluate the antibacterial synergy of synthetic CBD in combination with polymyxin B against a panel of polymyxin-resistant and -susceptible Gram-negative pathogens and elucidate, for the first time, the potential mechanism of the bacterial killing effects of polymyxins enhanced by CBD against *A. baumannii*, using untargeted metabolomics. The presented findings underlie the effective synergy and clinical potential of the polymyxin B–CBD combination for the treatment of MDR polymyxin-resistant Gram-negative infections.

## 2. Materials and Methods

### 2.1. Bacterial Isolates

Ten different *A. baumannii* isolates, including 5 polymyxin B-susceptible and 5 polymyxin B-resistant strains; 10 different *K. pneumoniae* isolates, including 6 polymyxin B-susceptible and 4 polymyxin B-resistant strains; and 7 different *P. aeruginosa* isolates, including 3 polymyxin B-susceptible and 4 polymyxin B-resistant strains (Table 1), were employed in this study.

### 2.2. Determination of MIC and FIC

The MICs of polymyxin B and CBD were determined for all bacterial isolates in triplicate on separate days using broth microdilution checkerboard assays. The MIC is defined as the lowest concentration of antimicrobial agent that shows a complete inhibition of visible bacterial growth after overnight incubation. The polymyxin B and CBD stock solutions were prepared immediately before each experiment. Polymyxin B (Betapharma, Shanghai, China) powder was dissolved in MilliQ water and sterilized by membrane syringe filters with a pore size of 0.22 micron (ThermoFisher Scientific, Melbourne, VIC, Australia). Cannabidiol (CBD) (Cayman Chemicals, Ann Arbor, Michigan, USA) was dissolved in dimethyl sulfoxide (DMSO; Sigma-Aldrich, Melbourne, VIC, Australia). Serial concentrations of DMSO (0.25%, 0.5%, 1%, 2.5% *v*/*v*) showed no inhibitory effect against all bacteria tested. Serial dilutions (2-fold) of the polymyxin B and CBD were prepared in a cation-adjusted Mueller–Hinton broth (CAMHB; Oxoid, Basingstoke, UK) to obtain solutions with various concentrations. The experiments were performed in 96-well microtiter plates (Techno Plas, Adelaide, SA, Australia) in broth culture containing ~10^6^ CFU/mL of the bacteria tested. The microdilution plates were incubated at 37 °C for 18–20 h.

The synergy tests for the combination were conducted using the fractional inhibitory concentration index (FICI) analysis. The formula used to calculate the FIC values was as follows: FICI = (MIC of drug A in combination ÷ MIC of drug A alone) + (MIC of drug B in combination ÷ MIC of drug B alone). The FICI results were defined as: synergism FIC < 0.5; addition FIC = 0.5–1.0; indifference FIC = 1–4; antagonism FIC ≥ 4 [22]. An MIC of 128 µg/mL for CBD was used to calculate the FICI scores.

### 2.3. Static Time-Kill Studies

Based on FIC results, static time-kill assays of polymyxin B monotherapy and in combination with CBD were performed against selected strains from different species. Prior to each time-kill assay, a sub-culture of the selected bacterial isolate was prepared from its stock cultures and incubated at 37 °C for 18~24 h. To prepare overnight culture, a single colony was transferred from nutrient agar plate to inoculate 10 mL of fresh CAMHB (Oxoid, UK) in a 50 mL Falcon tube (ThermoFisher Scientific, Australia) and incubated overnight (~16 h) in a water bath shaker at a temperature of 37 °C and 150 rpm shaking speed. Subsequently, the log phase was induced by inoculating 100 µL from the overnight culture into 10 mL of fresh CAMHB broth and incubated at 37 °C in a water bath shaker (shaking speed, 150 rpm) for 2 h. A 200 µL aliquot of bacterial suspension was inoculated into 4 borosilicate culture tubes (capacity 50 mL) containing 20 mL (1:100 dilution) of fresh CAMHB each. Three treatment tubes containing a starting culture of bacteria were spiked with the desired concentrations of polymyxin B and CBD monotherapies or in combination. One culture tube was drug-free to represent the control. At 0, 1, 4, 8 and 24 h, samples were taken from each tube and serially diluted in 0.9% saline, and then automatically plated onto nutrient agar plates using a Whitley Automated Spiral Plater (WASP). After 24 h incubation at 37 °C, the number of colonies on the plates were counted, and the time-kill graphs were plotted as time (hours) vs. log_10_ CFU/mL.

### 2.4. Bacterial Culture Preparation for Metabolomics

Single colonies of *A. baumannii* ATCC 19606 grown on a nutrient agar plate (Media Preparation Unit, University of Melbourne, Australia) were picked and inoculated into 10 mL of CAMHB (Oxoid, UK) in a 50 mL Falcon tube (ThermoFisher Scientific, Australia) and incubated at 37 °C overnight (~16 h) in a water bath shaker (150 rpm). The next day, a 100-fold dilution of bacterial culture was prepared by transferring a small amount of the overnight culture into 500 mL conical flasks (×4) containing fresh CAMHB. The flasks were immediately incubated in an incubator shaker (Bioline) at 37 °C with shaking at 180 rpm to log phase of ~10^8^ CFU/mL (OD600~0.5). Stock solutions of polymyxin B, CBD, or the combination were added accordingly to three treatment flasks with concentrations of 2 mg/L for polymyxin B, 4 mg/L for cannabidiol, and the combination of both compounds; the fourth flask served as control (drug-free). Samples were then taken at each time point (1 and 4 h) and immediately quenched to pause further bacterial metabolism. Subsequently, the OD600 reading for all samples was measured and normalized to ~0.5 with fresh CAMHB. The bacterial cells were pelleted with centrifugation at 3220× *g* at 4 °C for 10 min, and the supernatants were removed subsequently. The pellets were stored at −80 °C, awaiting metabolite extraction. Four biological replicates were prepared for each treatment condition to reduce the bias from inherent random variation.

### 2.5. Metabolite Extraction

The bacterial pellets were washed twice in 1 mL of 0.9% saline and centrifuged at 3220× *g* at 4 °C for 10 min. A cold extraction solvent (chloroform: methanol: water) containing 1 µM each of the internal standards (CHAPS, CAPS, PIPES and TRIS) was used to re-suspend the bacterial pellets. The pellets were then exposed to three freeze-thaw cycles (using liquid nitrogen) to release the intracellular metabolites. After the freezing and thawing process, the supernatants were obtained after centrifugation at 3220× *g* for 10 min at 4 °C and immediately transferred into an injection vial for liquid chromatography–mass spectrometry (LC–MS) analysis. An equal volume of each sample was combined and used as a quality control (QC) [40].

### 2.6. Data Processing, Bioinformatics, and Statistical Analyses

The LC–MS data processing, bioinformatics and statistical analysis were performed as we have previously described [41].

## 3. Results

### 3.1. Synergy Testing of Polymyxin B–Cannabidiol Combinations (FICI Calculation)

The synergistic antibacterial activity of polymyxin B and CBD as monotherapies and in combination was tested against a panel of clinical isolates of *A. baumannii*, *P. aeruginosa*, and *K. pneumoniae* (Table 1). The polymyxin B and CBD combinations demonstrated synergistic antibacterial activity against all of the tested strains of the three species *A. baumannii*, *P. aeruginosa*, and *K. pneumoniae* except for one *A. baumannii* (the LPS-deficient A. baumannii FADDI-AB065, indifference FICI = 2) and one *P. aeruginosa* (*P. aeruginosa* FADDI-PA019m, additive FICI = 0.53) (Table 1). On the other hand, polymyxin B and CBD monotherapies were found to be ineffective (MICs 8 to >128 mg/L) against the polymyxin B-resistant isolates (except for *A. baumannii* FADDI-AB065, CBD MIC = 0.5 mg/L) (Table 1).

### 3.2. Static Time-Kill Studies

**Figure 1:**
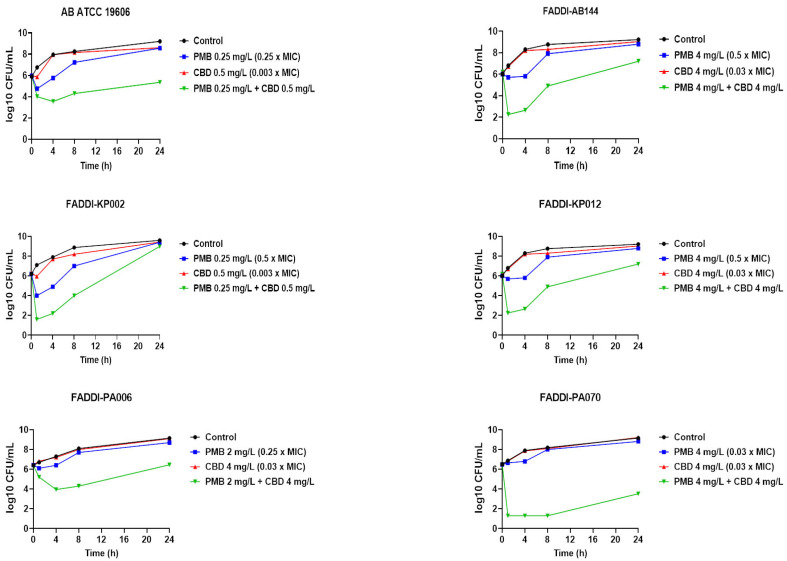
Time-kill curves for polymyxin B (PMB) and cannabidiol (CBD) monotherapies and in combinations against polymyxin B-susceptible *A. baumannii* strain ATCC 19606 (polymyxin B MIC = 1 mg/L, cannabidiol MIC > 64 mg/L) and polymyxin B-resistant *A. baumannii* strain FADDI-AB144 (polymyxin B MIC = 8 mg/L, cannabidiol MIC > 64 mg/L); against polymyxin B-susceptible *K. pneumoniae* FADDI-KP002 (polymyxin B MIC = 0.5 mg/L, cannabidiol MIC > 64 mg/L) and polymyxin B-resistant *K. pneumoniae* FADDI-KP012 (polymyxin B MIC = 32 mg/L, cannabidiol MIC > 64 mg/L); against polymyxin B-resistant *P. aeruginosa* strains FADDI-PA070 (polymyxin B MIC = 128 mg/L, cannabidiol MIC > 64 mg/L) and FADDI-PA006 (polymyxin B MIC = 8 mg/L, cannabidiol MIC > 64 mg/L). Data are mean values of three independent cultures, and vertical bars represent the standard deviations. Error bars are too small to appear in the graphs.

The time-kill assessment of polymyxin B and CBD as monotherapies or in combination was further carried out against polymyxin B-susceptible *A. baumannii* strain ATCC 19606 (polymyxin B MIC = 1 mg/L, CBD MIC > 64 mg/L) and polymyxin B-resistant *A. baumannii* strain FADDI-AB144 (polymyxin B MIC = 8 mg/L, CBD MIC > 64 mg/L); polymyxin B-resistant *P. aeruginosa* strains FADDI-PA070 (polymyxin B MIC = 128 mg/L, CBD MIC > 64 mg/L) and FADDI-PA006 (polymyxin B MIC = 8 mg/L, CBD MIC > 64 mg/L); and polymyxin B-susceptible *K. pneumoniae* strain FADDI-KP002 (polymyxin B MIC = 0.5 mg/L, CBD MIC > 64 mg/L) and polymyxin B-resistant *K. pneumoniae* strain FADDI-KP012 (polymyxin B MIC = 32 mg/L, CBD MIC > 64 mg/L) (Figure 1).

Polymyxin B monotherapy displayed significant killing activity against *A. baumannii* ATCC 19606, as manifested by a >2 log_10_ CFU/mL decline in bacterial counts compared with the control (untreated) at early time points (1 and 4 h); however, the killing curve of polymyxin B was comparable with the control at 24 h. Insignificant killing effect against the same strain was observed following CBD monotherapy across all time points (Figure 1). On the other hand, polymyxin B–CBD combination showed a higher killing curve compared to the control at all time exposures (>−2 log_10_ CFU/mL, 1 h; ≥−4 log_10_ CFU/mL, 4 and 8 h; >−3.5 log_10_ CFU/mL, 24 h). No significant decrease in the bacterial burden was noticed following polymyxin B (except at 4 h, −2 log_10_ CFU/mL) and CBD monotherapy against FADDI-AB144 at all time points, whilst a more than 4 log_10_ CFU/mL decrease in bacterial count compared with control was seen after the combination at 1 and 4 h (Figure 1). The combination also continued to reduce the bacterial burden by ≥2 log_10_ CFU/mL at later time points (8 and 24 h). Against *K. pneumoniae* strains, although a higher killing curve was observed after polymyxin B monotherapy (−3.0 log_10_ CFU/mL) against *K. pneumoniae* FADDI-KP002 at early time points (1 and 4 h), inconsistent regrowth occurred, with very minor log_10_ CFU/mL difference compared to the control after 24 h. Notably, the combination therapy was more effective in bacterial killing, as manifested by a ≥4.0–6.0 log_10_ CFU/mL decrease in bacterial counts compared with untreated controls across all time points (except 24 h). However, a completely different scene was observed against *K. pneumoniae* FADDI-KP012, wherein polymyxin B monotherapy did not display significant bacterial killing at all time points, as manifested by time-kill curves that resembled untreated controls (Figure 1). Nevertheless, polymyxin B and CBD combination therapy displayed potent bacterial killing effect, peaking at 1 and 4 h with a 3.0–6.0 log_10_ CFU/mL decline in bacterial counts compared to the control, which was sustained with a 3.0–4.0 log_10_ CFU/mL decline after 8 and 24 h, respectively, compared to the control (Figure 1). Regarding *P. aeruginosa* strains, only the combination therapy was effective in causing significant bacterial killing activity, as manifested by a >1.5–3 log_10_ CFU/mL (FADDI-PA006) and >5–6 log_10_ CFU/mL (FADDI-PA070) decrease in the bacterial burden across all time exposures (Figure 1). CBD monotherapy displayed no antimicrobial activity against any of the strains assessed during 24 h treatment, which was indicated by a time-kill curve pattern similar to the untreated control (Figure 1).

The current findings suggest that the synergy between polymyxin B and the CBD most likely arises from bioavailability synergy [42], which means that polymyxins permeabilize the Gram-negative OM and thereby allow the entry of CBD into the intracellular compartment to subsequently exert its action on its intracellular targets [36,38].

### 3.3. Metabolomics Analysis of A. baumannii ATCC 19606 Treated with Polymyxin B, Cannabidiol, and Their Combination

A total of 1127 putatively acquired metabolites were obtained; the highest number of metabolites (except for undefined class) belonged to lipid metabolism (268), followed by peptide metabolism (180) and amino acid metabolism (175). Nucleotides (41), carbohydrates (30), cofactor and vitamins (30), secondary metabolites (15), and glycans (3) were the least identified metabolite classes (Supplementary Figure S1).

The significant metabolites were determined by using one-way analysis of variance (ANOVA) (≥0.58-log_2_-fold; *p* ≤ 0.05). The data precision of all sample groups was agreeable at both time points (1 and 4 h), where the median RSDs were (17–23%) and (19–26%) for untreated (control) and treated samples, respectively, at all time points, consistent with some baseline dynamic variations in bacterial metabolism (Supplementary Table S1). The PCA plots demonstrated that the combination treatment samples were significantly distinguished from the untreated control samples, moderately at 1 h and largely at 4 h (Supplementary Figure S2A). Notably, the polymyxin B and combination treatments were nearly overlapped at 1 h, suggesting that polymyxin B initially governed the synergy of the combination treatment, whereas polymyxin B alone closely resembled the untreated control samples at the later time point, 4 h (Supplementary Figure S2A). CBD treated samples were undistinguished from the untreated control groups across all time exposures (1 and 4 h) (Supplementary Figure S2A). The same pattern of differences between the treated and untreated (control) groups was observed with the heatmaps (Supplementary Figure S2B).

A higher perturbation was induced by the combination treatment, as indicated by a perturbation of 267 (204 decreased and 63 increased) and 152 (139 decreased and 13 increased) significant metabolites at 1 and 4 h, respectively, whereas polymyxin B monotherapy perturbed 187 (174 decreased and 13 increased) significant metabolites at 1 h and a far smaller number at 4 h, with only 50 significantly decreased metabolites. CBD showed less effect, significantly perturbing only 29 (15 decreased and 14 increased) and 10 (6 decreased and 4 increased) significant metabolites at 1 and 4 h, respectively (Supplementary Figure S3A).

The metabolic profiling analysis of the significantly altered metabolites indicated that the combination treatment, at both time points, largely perturbed (mainly decreased) lipids, amino acids and peptides compared to the slightly impacted metabolite carbohydrates, nucleotides and cofactors and vitamin metabolism (Supplementary Figure S3B). Likewise, but to a lesser extent, polymyxin B monotherapy induced a similar pattern, wherein lipids, amino acids and peptides were the impacted metabolite classes (Supplementary Figure S3B). On the other hand, CBD monotherapy caused a suppression in the levels of lipid intermediates (mainly at 1 h) and an elevation in the levels of amino acids (mainly at 4 h) (Supplementary Figure S3B). Notably, the combination treatment induced 148 and 103 unique significant metabolites at 1 and 4 h, respectively (Supplementary Figure S3C). There were only 10 and 4 intermediates in common between all treatment conditions at 1 and 4 h, respectively (Supplementary Figure S3C). The combination treatment shared ~30–35% of the intermediates with polymyxin B monotherapy at 1 and 4 h (Supplementary Figure S3C).

#### 3.3.1. Analyses of Dysregulated Glycerophospholipid and Fatty Acid Metabolism

The major lipid components of the cellular envelope of Gram-negative bacteria include phosphatidylglycerol (PG), phosphatidylethanolamine (PE) and cardiolipin [43]. These lipid precursors are crucial to maintaining the integrity of the bacterial cell envelope to adapt and survive under ever-changing environmental stress [44].

**Figure 2:**
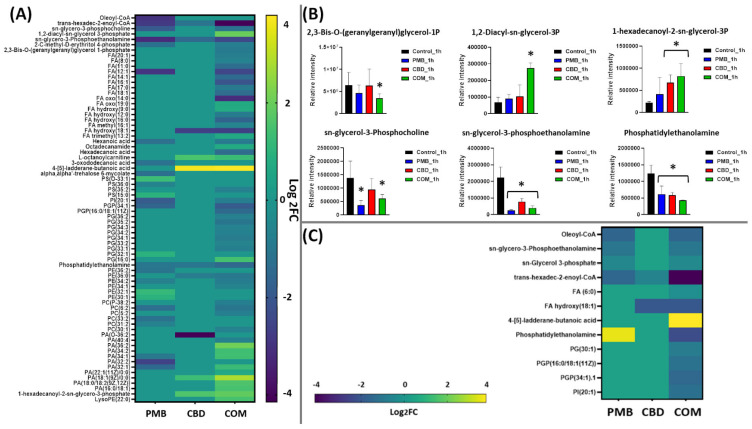
Perturbations of bacterial lipids. (**A**) Heatmap and (**B**) bar charts for significantly perturbed lipids in *A. baumannii* ATCC 19606 following treatment with polymyxin B (PMB, blue), cannabidiol (CBD, red) and the combination (COM, green) at 1 h. (**C**) Heatmap for significantly perturbed lipids in *A. baumannii* ATCC 19606 following treatment with polymyxin B (PMB, blue), cannabidiol (CBD, red) and the combination (COM, green) at 4 h. Lipid names are putatively assigned based on accurate mass (≥0.59-log_2_-fold, *p* ≤ 0.05). Control, untreated samples; PE, phosphoethanolamines; PG, glycerophosphoglycerols; PS, glycerophosphoserines; PC, glycerophosphocholines; PA, glycerophosphates; LysoPE, lysophosphatidylethanolamines; PGP, glycerophosphoglycerophosphates; FA, fatty acids. * ≥0.59-log_2_-fold, *p* ≤ 0.05 (one-way ANOVA).

At 1 h, polymyxin B–CBD combination therapy induced significantly more perturbations in all lipid classes, including fatty acids and glycerophospholipids (e.g., phosphatidylserine, PS; glycerophosphocholines, PC; phosphatidylethanolamine, PE; glycerophosphates, PA; and phosphatidylglycerol, PG) compared to the polymyxin B and CBD monotherapies (Figure 2A). The combination treatment caused more overrepresentation in the levels of glycerophospholipids compared to other lipid classes such as fatty acids. Notably, phosphatidylethanolamine (log_2_FC = −1.5), and 1-hexadecanoyl-2-*sn*-glycero-3-phosphate (synonym: 1-palmitoylglycerol 3-phosphate, log_2_FC = 1.8) were among the significantly impacted glycerophospholipids following the combination treatment at 1 h (Figure 2B). Furthermore, the abundance of principal constituents of bacterial membrane lipids such as 1,2-diacyl-*sn*-glycerol 3-phosphate (phosphatidic acid) (log_2_FC = 1.9), *sn*-glycero-3-phosphocholine (log_2_FC = −1.1), *sn*-glycero-3-phosphoethanolamine (log_2_FC = −2.4), 2,3-Bis-O-(geranylgeranyl)glycerol 1-phosphate (log_2_FC = −0.88), and trans-hexadec-2-enoyl-CoA (log_2_FC = −4.1) were significantly altered following combination treatment (Figure 2A,B) [45,46]. Similarly, fundamental building blocks of membrane lipids as well as the lipid A part of lipopolysaccharides, fatty acids, underwent significant perturbations following combination treatment, including hexadecanoic acid (palmitic acid, log_2_FC = −1.4), FA(16:1) (log_2_FC = −2.4), FA(14:1) (log_2_FC = −1.4) and FA(17:0) (log_2_FC = −0.88) (Figure 2A) [47,48].

The impact of polymyxin B monotherapy was far less than that from the combination treatment at 1 h, and glycerophospholipids experienced a greater perturbation compared to fatty acids (Figure 2A). Importantly, the main precursors of bacterial lipid membrane, which are involved in fatty acid elongation and assembly of glycerolipids, were significantly altered following polymyxin B monotherapy, namely oleoyl-CoA, trans-hexadec-2-enoyl-CoA, phosphatidylethanolamine, *sn*-glycero-3-phosphocholine and *sn*-glycero-3-phosphoethanolamine (≥−1.0-log_2_-fold; *p* ≤ 0.05) (Figure 2A,B). Notably, although cannabidiol caused marginal effect on lipid metabolism at 1 h, it induced a significant perturbation in the levels of major lipid intermediates of bacterial membrane, such as phosphatidylethanolamine (log_2_FC = −1.0), trans-hexadec-2-enoyl-CoA (log_2_FC = −1.1), *sn*-glycero-3-phosphoethanolamine (log_2_FC = −1.5) and 1-hexadecanoyl-2-*sn*-glycero-3-phosphate (log_2_FC = 1.5) (Figure 2A,B).

On the other hand, at 4 h, although the impact of combination therapy on lipid metabolism declined, a remarkable perturbation pattern of lipid intermediates particularly against glycerophospholipids was observed (Figure 2C). The levels of two essential lipids (*sn*-glycerol 3-phosphate and *sn*-glycero-3-phosphoethanolamine) involved in the rate-limiting step of the biosynthesis of bacterial membrane phosphatidic acid declined in response to the combination therapy (≥−1.0-log_2_-fold; *p* ≤ 0.05) (Figure 2C) [45]. Furthermore, the effect on fatty acid elongation was slightly sustained following the combination therapy, as manifested by a significant decline in the abundance of oleoyl-CoA (log_2_FC = −2.8) (Figure 2C).

In comparison, the impact from polymyxin B and cannabidiol monotherapies at 4 h started to disappear; polymyxin B decreased the abundance of oleoyl-CoA, *sn*-glycerol 3-phosphate and *sn*-glycero-3-phosphoethanolamine (≥−1.0-log_2_-fold; *p* ≤ 0.05), whereas the level of only one fatty acid precursor significantly declined after cannabidiol treatment (Figure 2C).

#### 3.3.2. Analyses of Dysregulated Amino-Sugar and Nucleotide-Sugar Metabolism, the Pentose Phosphate Pathway, and Downstream Peptidoglycan and Lipopolysaccharide Biosynthesis

**Figure 3:**
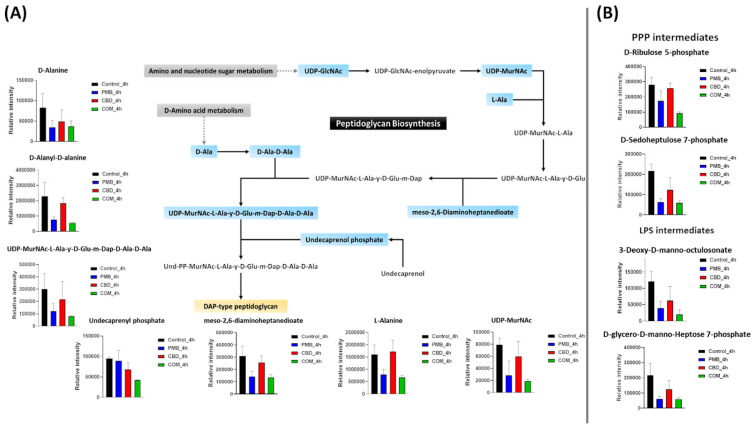
(**A**) Schematic diagram depicting significantly impacted metabolites and bar charts of amino-sugar and nucleotide-sugar metabolism and downstream peptidoglycan biosynthesis for *A. baumannii* ATCC 19606 treated with polymyxin B (PMB) or cannabidiol (CBD) monotherapy and the combination (COM) after 4 h exposure. (**B**) Bar charts for the significantly impacted metabolites of pentose phosphate pathway (PPP) and lipopolysaccharide (LPS) biosynthesis following polymyxin B (PMB) or cannabidiol (CBD) monotherapy and the combination treatment at 4 h (≥0.59-log_2_-fold, *p* ≤ 0.05). Blue rectangles: significantly inhibited metabolites; Red rectangles: Significantly increased metabolites.

Gram-negative bacteria are surrounded by a complex multilayered structure (OM, a thin peptidoglycan cell wall, and inner cell membrane) that protects them from the harsh environment, but selectively enables the passage of nutrients from the outside and waste products from the inside [49]. Notably, polymyxin B–cannabidiol treatment caused a significant perturbation (slightly at 1 h and largely at 4 h) in the abundance of key metabolites involved in amino-sugar and nucleotide-sugar metabolism, the pentose phosphate pathway (PPP), and the direct interrelated metabolic pathways of cell envelope biogenesis, peptidoglycan, and lipopolysaccharide (LPS) formation (Figure 3; Supplementary Figure S4). At 1 h, the abundance of one essential component (UDP-N-acetyl-D-glucosamine (UDP-GlcNAc)) of amino-sugar and nucleotide-sugar metabolism and two fundamental precursors of PPP (D-sedoheptulose 7-phosphate and D-ribulose 5-phosphate (Ru5P)) underwent a significant decline after the combination treatment (≥−2.0-log_2_-fold; *p* ≤ 0.05) (Supplementary Figure S4). As a downstream consequence, the combination treatment also caused a marked depletion in the abundance of three peptidoglycan building blocks, namely UDP-N-acetylmuramoyl-L-alanyl-D-glutamyl-*meso*-2,6-diaminopimeloyl-D-alanyl-D-alanine (UDP-MurNAc-L-Ala-ƴ-D-Glu-*m*-Dap-D-Ala-D-Ala, log_2_FC = −3.2), D-alanyl-D-alanine (log_2_FC = −5.7), and *meso*-2,6-diaminoheptanedioate (log_2_FC = −2.2) (Supplementary Figure S4). Consequently, a dramatic decrease in the levels of two common intermediates (3-deoxy-D-manno-octulosonate (KDO), log_2_FC = −1.2; D-glycero-D-manno-heptose 7-phosphate, log_2_FC = −2.5) of LPS formation was observed following the combination treatment at 1 h (Supplementary Figure S4).

On the other hand, at 1 h, although polymyxin B monotherapy caused a significant alteration in the main pathways involved in cell envelope assembly, its effect was less potent compared to that of the combination therapy (Supplementary Figure S4). The levels of one amino-sugar and nucleotide-sugar intermediate, UDP-GlcNAc, and two intermediates of PPP (D-sedoheptulose 7-phosphate and D-ribulose 5-phosphate) experienced a substantial decline after polymyxin B monotherapy (≥−0.59-log_2_-fold; *p* ≤ 0.05) (Supplementary Figure S4). Simultaneously, polymyxin B monotherapy also disrupted peptidoglycan biosynthesis, as manifested by an alteration in the levels of UDP-MurNAc-L-Ala-ƴ-D-Glu-*m*-Dap-D-Ala-D-Ala (log_2_FC = −2.0), D-alanyl-D-alanine (log_2_FC = −2.0), and *meso*-2,6-diaminoheptanedioate (log_2_FC = −1.5) (Supplementary Figure S4). Like the combination treatment, albeit to a lesser extent, polymyxin B alone induced a marked decrease in the abundance of two essential inner-core oligosaccharide moieties of LPS, namely 3-deoxy-D-manno-octulosonate and D-glycero-D-manno-heptose 7-phosphate (≥−0.59-log_2_-fold; *p* ≤ 0.05) (Supplementary Figure S4). Cannabidiol monotherapy induced far less effect on the pathways of bacterial cell envelope formation, wherein the abundance of only UDP-GlcNAc, an amino-sugar and nucleotide-sugar intermediate, was considerably reduced after cannabidiol monotherapy (≥−0.59-log_2_-fold; *p* ≤ 0.05) (Supplementary Figure S4).

At 4 h, different patterns of perturbation were observed wherein the combination treatment induced a profound inhibitory impact on amino-sugar and nucleotide-sugar metabolism and PPP, peptidoglycan and LPS biosynthesis (Figure 3A,B). The levels of two important building blocks of amino-sugar and nucleotide-sugar metabolism, namely UDP-GlcNAc and UDP-N-acetylmuramate (UDP-MurNAc), were depleted after the combination treatment at 4 h (≥−2.0-log_2_-fold; *p* ≤ 0.05) (Figure 3A). Similarly to the combination impact at 1 h, the abundance of two crucial PPP precursors (D-sedoheptulose 7-phosphate and D-ribulose 5-phosphate) was significantly decreased following the combination therapy (≥−1.5-log_2_-fold; *p* ≤ 0.05) (Figure 3B). A greater impact on the downstream pathway for peptidoglycan biosynthesis was seen after the combination therapy. This was shown by the fact that the combination markedly decreased the abundance of five peptidoglycan biosynthesis intermediates, namely UDP-MurNAc-L-Ala-ƴ-D-Glu-*m*-Dap-D-Ala-D-Ala (log_2_FC = −1.9), D-alanine (log_2_FC = −1.5), D-alanyl-D-alanine (log_2_FC = −2.0), L-alanine (log_2_FC = −1.2) and *meso*-2,6-diaminoheptanedioate (log_2_FC = −1.2) (Figure 3A). Importantly, the combination treatment caused a profound decrease (log_2_FC = −1.1) in the abundance of undecaprenyl phosphate (C55-P) at 4 h (Figure 3A). The combination treatment also reduced the abundance of two essential components of LPS biogenesis, KDO and D-glycero-D-manno-heptose 7-phosphate (log_2_FC = −2.5 and 1.8, respectively) (Figure 3B).

Although polymyxin B alone perturbed a similar number (except for undecaprenyl phosphate) of intermediates involved in the cell envelope biosynthetic interconnected pathways, it was relatively less potent than the combination therapy at 4 h (Figure 3A,B). This was manifested by a lower intensity reduction in the levels of two amino-sugar and nucleotide-sugar metabolism intermediates (UDP-GlcNAc and UDP-MurNAc) and an equal number of PPP components (D-sedoheptulose 7-phosphate and Ru5P) due to polymyxin B monotherapy (≥−0.59-log_2_-fold; *p* ≤ 0.05) (Figure 3A,B). Consequently, the abundance of five essential peptidoglycan intermediates was diminished after polymyxin B monotherapy, including UDP-MurNAc-L-Ala-ƴ-D-Glu-*m*-Dap-D-Ala-D-Ala (log_2_FC = −1.2), D-alanine (log_2_FC = −1.2), D-alanyl-D-alanine (log_2_FC = −1.5), L-alanine (log_2_FC = −1.0) and *meso*-2,6-diaminoheptanedioate (log_2_FC = −1.1) (Figure 3A). The impact of polymyxin B monotherapy on the LPS biosynthetic pathway was sustained at 4 h, although it was less potent compared to the combination therapy, causing a less intense reduction in the abundance of KDO and D-glycero-D-manno-heptose 7-phosphate (log_2_FC = −1.6, −1.7, respectively) (Figure 3B). Intriguingly, at 4 h, the inhibitory impact of cannabidiol monotherapy on the amino sugar donor, UDP-GlcNAc, continued, and moreover, it was also effective in decreasing the level of a key inner-core LPS precursor, namely KDO (≥−0.59-log_2_-fold; *p* ≤ 0.05) (Figure 3A,B).

#### 3.3.3. Analyses of Perturbations in Nucleotide and Peptide Metabolism

Bacterial RNA synthesis requires ATP, guanosine-triphosphate (GTP), cytidine-triphosphate (CTP) and uridine-triphosphate (UTP) as the building blocks, and deoxynucleotide triphosphates (such as dATP, dCTP and dGTP) are the substrates for bacterial DNA polymerases, which are responsible for both replication and repair of cellular DNA [50,51].

**Figure 4:**
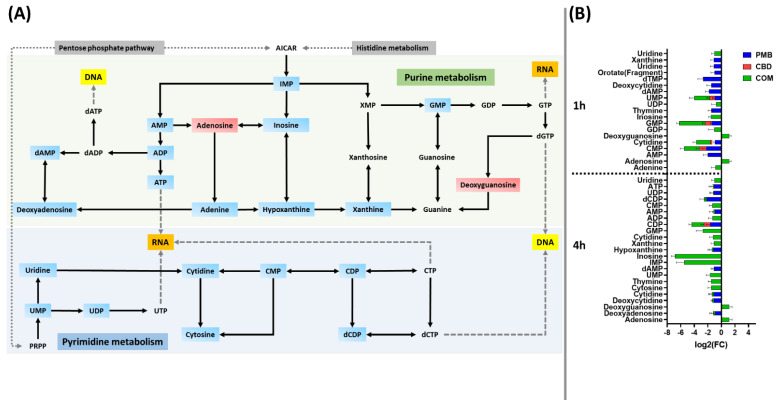
(**A**) Graph showing the impacted pyrimidine and purine metabolism for *A. baumannii* ATCC 19606 after 4 h treatment with polymyxin B (PMB), cannabidiol (CBD) and their combination (COM). (**B**) Bar charts for the significantly impacted metabolites of pyrimidine and purine metabolism after treatment with polymyxin B (PMB) or cannabidiol (CBD) monotherapy and their combination (COM) at 1 and 4 h (≥0.59-log_2_-fold, *p* ≤ 0.05). Blue rectangles: significantly inhibited metabolites; Red rectangles: Significantly increased metabolites.

Polymyxin B monotherapy caused a marked depletion in the levels of nucleotides predominantly at 1 h and to a lesser extent at 4 h, and this effect was even more pronounced following combination treatment particularly at 4 h (≥0.59-log_2_-fold; *p* ≤ 0.05) (Figure 4A,B). At 1 h, a marked decline in the levels of 14 nucleotides was observed following polymyxin B monotherapy, including dAMP, dTMP, uridine and xanthine (≥−1.0-log_2_-fold; *p* ≤ 0.05) (Figure 4B), whereas cannabidiol monotherapy had modest effects, significantly decreasing the abundance of five fundamental nucleotides at 1 h (≥−0.59-log_2_-fold; *p* ≤ 0.05) (Figure 4B). Fifteen nucleotides underwent a remarkable decrease in their levels after combination treatment (except for an increment in adenosine and deoxyguanosine levels) (Figure 4B).

At 4 h, the combination profoundly perturbed the abundance of 24 nucleotide intermediates (≥1.0-log_2_-fold; *p* ≤ 0.05) (Figure 4A,B). Among the nucleotides, IMP, inosine, adenosine, and deoxyguanosine were uniquely perturbed due to the combination therapy at both time points (1 and 4 h) (≥1.0-log_2_-fold; *p* ≤ 0.05) (Figure 4B). Similarly to the 1 h time point, albeit to a lesser extent, polymyxin B monotherapy caused a reduction of 10 nucleotides at 4 h (≥−0.59-log_2_-fold; *p* ≤ 0.05) (Figure 4B), whereas cannabidiol monotherapy displayed a negligible effect at 4 h by depleting only the CDP level (log_2_FC = −0.94) (Figure 4B).

Notably, a large number of metabolites involved in peptide metabolism were significantly altered following combination treatment at 1 h (41 peptides) and to a limited degree at 4 h (17 peptides) (≥0.59-log_2_-fold, *p* ≤ 0.05) (Supplementary Figure S5). Likewise, but to a lesser extent, polymyxin B monotherapy caused a significant inhibitory effect on 34 precursors of peptide metabolism at 1 h, while only two peptide intermediates showed a significant decrease following polymyxin B monotherapy at 4 h (Supplementary Figure S5). No significant effect was observed in the samples treated with cannabidiol monotherapy at both time exposures (1 and 4 h).

## 4. Discussion

To prolong and maximize the clinical efficacy of polymyxins and prevent the emergence of antibiotic resistance, novel polymyxin combination therapy against MDR Gram-negative bacteria such as *A. baumannii* is desperately needed. Although researchers have shown that polymyxins in combination with cannabidiol displayed synergistic killing activity against Gram-negative bacteria, including *A. baumannii*, they did not perform time-kill assays or investigate the precise mechanisms behind the synergistic killing activity of polymyxins in combination with the cannabidiol against *A. baumannii* [33,35,36]. To the best of our knowledge, this is the first study to decipher the mechanisms of synergistic killing activity of polymyxins and synthetic cannabidiol combination therapy against *A. baumannii* using untargeted metabolomics.

The checkerboard and time-kill assays demonstrated synergistic bacterial killing by polymyxins plus cannabidiol treatment against a panel of MDR Gram-negative pathogens (*A. baumannii*, *K. pneumoniae* and *P. aeruginosa*) (Figure 1; Table 1). The metabolomics study showed that polymyxin B–cannabidiol treatment caused a greater perturbation in *A. baumannii* ATCC 19606 metabolome compared to monotherapies, particularly at the later time exposure (4 h). The most significant findings for integrating pathway enrichment analysis include (i) dysregulation in bacterial cell envelope biogenesis, (ii) inhibition of DNA and RNA metabolism, as reflected by the major perturbation of purine and pyrimidine metabolism, and (iii) perturbation of peptide metabolism.

Polymyxins exert primary antibacterial killing activity by disorganizing the bacterial OM [9,52]. Unsurprisingly, at 1 h, polymyxin B caused a marked suppression, albeit far less compared to that achieved with the combination, in fatty acids, glycerophospholipids metabolism as well as key lipids of OM such as trans-hexadec-2-enoyl-CoA, *sn*-glycero-3-phosphocholine and *sn*-glycero-3-phosphoethanolamine (Figure 2A,B). Our findings agreed with previous metabolomics and transcriptomics studies that showed that *A. baumannii* metabolites and genes involved primarily in bacterial OM biosynthesis and phospholipid trafficking experienced a significant alteration at 1 h polymyxin posttreatment [19,53]. However, the impact from polymyxin B monotherapy on levels of essential bacterial membrane lipids was minimal at 4 h, suggesting that polymyxin resistance may occur within 4 h after polymyxin B monotherapy. The antimicrobial effect of cannabidiol has recently gained great attention; however, the precise mode of action is still unclear [34,35,36,37,38]. It was found that cannabidiol inhibited membrane lipid synthesis of *S. aureus* [38]. This evidence is in line with our findings that showed the inhibitory impact of cannabidiol on levels of essential bacterial membrane lipids at 1 h, including phosphatidylethanolamine, *sn*-glycero-3-phosphoethanolamine and 1-hexadecanoyl-2-*sn*-glycero-3-phosphate (Figure 2A). Nevertheless, this effect disappeared at the later time exposure of 4 h. Promisingly, the combination caused extensive perturbations (largely at 1 h and to a lesser extent at 4 h) in fatty acids and glycerophospholipids (Figure 2A,B). There has been growing interest in fatty acid biosynthesis as a major target for antibiotic development, for instance, the newly discovered fatty acid elongation inhibitors, platensimycin and platencin [54]. A greater reduction in the levels of key bacterial membrane lipids (e.g., phosphatidylethanolamine, 1,2-diacyl-*sn*-glycerol 3-phosphate (phosphatidic acid), *sn*-glycero-3-phosphoethanolamine and *sn*-glycerol 3-phosphate) was observed after the combination treatment at both time points (1 and 4 h) (Figure 2A,B). In Gram-negative bacteria, phosphatidylethanolamine accounts for 70–80% of total membrane lipids, which are synthesized on the cytoplasmic side of the inner membrane [55,56]. It has been found that an alteration in the levels of phosphatidylethanolamine leads to critically compromised cell integrity and ultimately cell death [44]. *Sn*-glycerol 3-phosphate is an essential precursor required for the formation of membrane phospholipids; it undergoes acylation at the 1-position to form lysophosphatidic acid (LPA), and then a second acylation step produces phosphatidic acid (PA), the principal component in the synthesis of bacterial membrane glycerolipids [57]. Targeting phosphatidic acid synthesis in Gram-negative bacteria might offer a new and effective strategy to develop novel antibacterial therapeutics [46].

Importantly, our study is the first to report that combining polymyxin B with cannabidiol caused a marked suppression in the components of peptidoglycan and LPS biogenesis at 1 h, including UDP-MurNAc-L-Ala-ƴ-D-Glu-*m*-Dap-D-Ala-D-Ala and D-alanyl-D-alanine (Supplementary Figure S4). This influence may have arisen from inhibition of amino-sugar and nucleotide-sugar metabolism and the pentose phosphate pathway, which are key sources of precursors for peptidoglycan and LPS synthesis. This was manifested by a significant decline in the levels of UDP-GlcNAc, D-sedoheptulose 7-phosphate and Ru5P after combination treatment (Supplementary Figure S4). UDP-GlcNAc plays a pivotal role as an amino sugar donor in several transferase reactions in the biosynthesis of peptidoglycan, the core lipid A components of the LPS, and certain *O*-antigens of Gram-negative bacteria [58,59]. The synthesis of KDO, an essential residue of the LPS inner core, is initiated by the enzyme D-arabinose-5-phosphate isomerase (API), which regulates the reversible isomerization of D-ribulose 5-phosphate (Ru5P) to D-arabinose-5-phosphate, a precursor of 3-deoxy-D-manno-octulosonate [60]. It is important to note that the combination treatment remained effective in disorganizing the bacterial envelope formation at 4 h, in which more perturbations in peptidoglycan and LPS biosynthesis were most prominent (Figure 3A,B). Intriguingly, the combination treatment caused a profound decrease in the abundance of undecaprenyl phosphate (C55-P), also known as bactoprenol, a sugar carrier lipid that mediates the biosynthesis of bacterial extracellular polysaccharides such as cell wall peptidoglycan and LPS [61,62], at 4 h (Figure 3A,B). It has been proposed that bactoprenol is a potential novel target for new antibacterial agents [63,64].

Consistent with our previous results, it was not unusual that polymyxin B monotherapy caused a significant alteration, though less intense compared to the combination, in the main pathways involved in the cell envelope assembly, including peptidoglycan and LPS biosynthesis at both time points (1 and 4 h) (Figure 3A,B) [17,18,19]. Remarkably, pathway analysis also showed that despite being a non-antibiotic, cannabidiol reduced the KDO and UDP-GlcNAc levels at 4 h (Figure 3B). Hence, taking data from all of the above-mentioned studies together, a possible mechanism of synergistic killing by the polymyxin B–cannabidiol combination is strongly related to the suppression of amino sugar and nucleotide sugar metabolism and PPP and subsequent peptidoglycan and LPS biosynthesis, which eventually leads to bacterial membrane structure deformity.

Apart from its disruptive impact on the bacterial membrane, the combination therapy produced profound changes in the nucleotide pool of *A. baumannii* ATCC 19606 at both time exposures (1 and 4 h); however, the maximum effect on the nucleotide pool was at 4 h (Figure 4A,B). The perturbed nucleotides were mainly those related to purine and pyrimidine metabolism (e.g., ATP, ADP, UDP and GMP), which ultimately impacted DNA and RNA synthesis. Nucleotide metabolism such as purine metabolism is actively involved in antibiotic efficacy and lethality [65,66]. It has been found that dysregulation in purine metabolism can alter antibiotic lethality; this has been manifested by an increase in the bacterial killing activity of gentamicin following genetic deletion of enzymes involved in purine metabolism [65]. Given the significant alteration of the majority of the nucleotides involved in purine and pyrimidine, it is highly likely that the combination treatment compromised DNA and RNA biosynthesis in *A. baumannii*. Notably, it has been previously reported that polymyxins were able to dysregulate the nucleotide pool of different Gram-negative pathogens such as *A. baumannii*, *K. pneumoniae* and *P. aeruginosa* [50,64,65]. A recent study using radiolabeled macromolecular synthesis assays revealed the ability of cannabidiol to inhibit DNA, RNA and protein synthesis in *S. aureus* [38]. In line with these findings, polymyxin B and cannabidiol monotherapies demonstrated significant perturbations, albeit to a lesser extent compared to their combination, in the levels of nucleotides at 1 h, which then faded away at 4 h (Figure 4A,B). In combination, our findings showed polymyxin B and cannabidiol additionally affected peptide metabolism, which most likely led to inhibition of protein synthesis in *A. baumannii*.

Overall, the novel findings from this study are that polymyxin B monotherapy produces significant changes in multiple cellular metabolic pathways of cell envelope biogenesis, all of which were further disorganized by adding cannabidiol. Furthermore, the combination therapy greatly perturbed the nucleotide and peptide pools, suggesting a unique mechanism (non-bacterial membrane involvement) of synergistic killing for the polymyxin B–cannabidiol treatment. The current findings also showed that cannabidiol affects LPS, DNA, and lipid biosynthesis. The study highlights the importance of elucidating the complex and dynamic interactions of multiple cellular metabolic pathways due to antibiotic–nonantibiotic treatment. Such findings will aid in the understanding of the most likely mechanisms behind the killing synergy and ultimately facilitate future repurposing of cannabidiol as an antimicrobial agent in combination with polymyxins.

## Figures and Tables

**Table 1:** Antimicrobial activity of polymyxin B (PMB) and cannabidiol (CBD) in monotherapy and in combination.

		MIC (mg/L)				FIC Index
Species	Polymyxin-Susceptible Isolates	PMB	CBD	FIC PMB	FIC CBD	PMB-CBD
*Acinetobacter baumannii*	ATCC 17978	0.25	>64	0.0625	0.25	0.25
	ATCC 19606	1	>64	0.0625	0.5	0.062
	FADDI-AB146	1	>64	0.125	0.5	0.12
	FADDI-AB150	2	>64	0.0625	0.5	0.03
	FADDI-AB151	2	>64	0.0625	0.25	0.03
	Polymyxin-resistant isolates					
	FADDI-AB144	8	>64	0.5	1	0.06
	FADDI-AB148	8	>64	0.25	1	0.03
	FADDI-AB143	16	>64	0.5	2	0.04
	FADDI-AB065	64	0.5	64	0.5	2.00
	FADDI-AB060	128	>64	4	2	0.04
*Klebsiella pneumoniae*	Polymyxin-susceptible isolates					
	Kp BM1	0.25	>64	0.0625	0.5	0.25
	FADDI-KP002	0.5	>64	0.0625	1	0.13
	FADDI-KP069	0.5	>64	0.0625	4	0.15
	KPATCC13883	0.5	>64	0.0625	2	0.14
	ATCC 700721	0.5	>64	0.125	2	0.26
	FADDI-KP005	1	>64	0.125	1	0.13
	Polymyxin-resistant isolates					
	FADDI-KP003	32	>64	4	4	0.15
	FADDI-KP012	32	>64	2	4	0.09
	FADDI-KP070	64	>64	2	4	0.06
	KPATCC13883R	128	>64	8	4	0.09
*Pseudomonas aeruginosa*	Polymyxin-susceptible isolates					
	FADDI-PA019 ma	1	>64	0.5	4	0.53
	FADDI-PA007 n/mb	1	>64	0.25	1	0.25
	FADDI-PA020 m	2	>64	0.25	8	0.18
	Polymyxin-resistant isolates					
	FADDI-PA006 m	8	>64	0.5	0.5	0.06
	FADDI-PA066 n/m	32	>64	2	4	0.04
	FADDI-PA070 n/m	128	>64	2	2	0.02
	FADDI-PA064 n/m	128	>64	8	8	0.12

m, mucoid; n/m, nonmucoid. FIC = FIC INDEX = (MIC polymyxin B in combination with cannabidiol/MIC polymyxin B monotherapy) + (MIC cannabidiol in combination with polymyxin B/MIC cannabidiol monotherapy); synergy FIC < 0.5; additivity FIC = 0.5–1.0; indifference FIC = 1–4; antagonism FIC ≥ 4 (not observed).

## Data Availability

All data available are reported in the article.

## Supplementary Materials

The following supporting information can be downloaded at: https://www.mdpi.com/article/10.3390/pharmaceutics14040786/s1, Figure S1: Total acquired metabolites and the proportion of each metabolite classes; Table S1: Data precision of individual samples represented as the median relative standard deviation (RSD) for all metabolites of *A. baumannii* ATCC 19606 based on all biological replicates (*n* = 4) of each group (*n* = 8 for technical replicates of PBQCs); Figure S2: (A) PCA plots for metabolite levels from *A. baumannii* ATCC 19606 samples treated with polymyxin B (PMB), cannabidiol (CBD), and their combination (COM) at 1 and 4 h. Each data set represents a total of 8 samples of 4 biological replicates of each condition. Purple = control; Cyan = polymyxin B (PMB); Red = cannabidiol (CBD); Green = combination. (B) Heatmap profiles of *A. baumannii* ATCC 19606 with hierarchical clustering of all identified metabolites after treatment with polymyxin B (PMB), cannabidiol (CBD), and their combination (COM) at 1 and 4 h; Figure S3: (A) Volcano plots show the total number of significant metabolites after antibiotic monotherapy and combination treatment of *A. baumannii* ATCC 19606 at 1 and 4 h. (B) The summary of significantly changed metabolites of *A. baumannii* ATCC 19606 from different categories following PMB and CBD monotherapies and their combination treatment at 1 and 4 h. Changes (≥0.59-log_2_-fold, *p* ≤ 0.05). (C) Venn diagrams showing the number of metabolites significantly affected by each treatment for *A. baumannii* ATCC 19606 at 1 and 4. Significant metabolites were selected with (≥0.59-log_2_-fold, *p* ≤ 0.05); Figure S4: The bar charts of significantly impacted metabolites of amino-sugar and nucleotide-sugar metabolism, PPP and downstream peptidoglycan and LPS biosynthesis for *A. baumannii* ATCC 19606 treated with polymyxin B (PMB) or cannabidiol (CBD) monotherapy and the combination (COM) after 1h exposure (≥1.0-log_2_-fold, *p* ≤ 0.05); Figure S5: The fold change charts of significantly impacted metabolites of peptides metabolism for *A. baumannii* ATCC 19606 treated with polymyxin B (PMB) or cannabidiol (CBD) monotherapy and the combination (COM) after 1 and 4h exposure (≥1.0-log_2_-fold, *p* ≤ 0.05).
