# Supplementary material for: Mechanisms Underlying Synergistic Killing of Polymyxin B in Combination with Cannabidiol against Acinetobacter baumannii: A Metabolomic Study
Source: Pharmaceutics. 2022 Apr 3;14(4):786. doi: 10.3390/pharmaceutics14040786 (PMC9025570; doi:10.3390/pharmaceutics14040786)
Supplement: Supplementary file 1 [file pharmaceutics-14-00786-s001.zip › pharmaceutics-1611562-supplementary.pdf]

pharmaceutics

MDPI

Supplementary Materials: Mechanisms Underlying Synergistic Killing of Polymyxin B in Combination with Cannabidiol Against *Acinetobacter baumannii*: A Metabolomic Study

Maytham Hussein, Rafah Allobawi, Irini Levou, Mark A T Blastovich, Gauri G. Rao, Jian Li, Tony Velkov

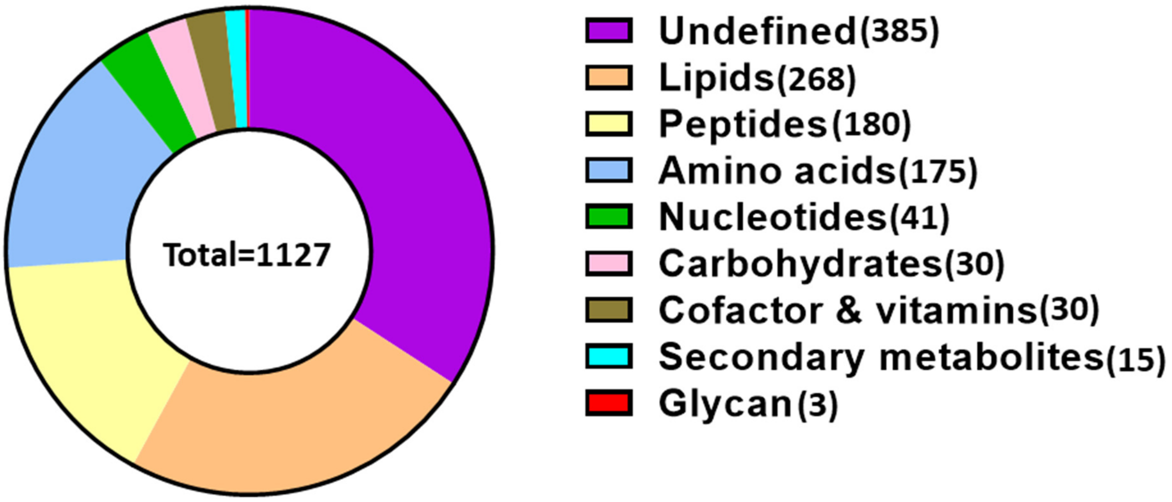

Figure S1. Total acquired metabolites and the proportion of each metabolite classes.

Table S1. Data precision of individual samples represented as the median relative standard deviation (RSD) for all metabolites of *A. baumannii* ATCC 19606 based on all biological replicates (n=4) of each group (n= 8 for technical replicates of PBQCs).

	**Median RSD %**	
	**1h**	**4h**
**Untreated Control**	17	23
**Polymyxin B**	23	26
Cannabidiol	19	24
**Combination**	20	24

(A)

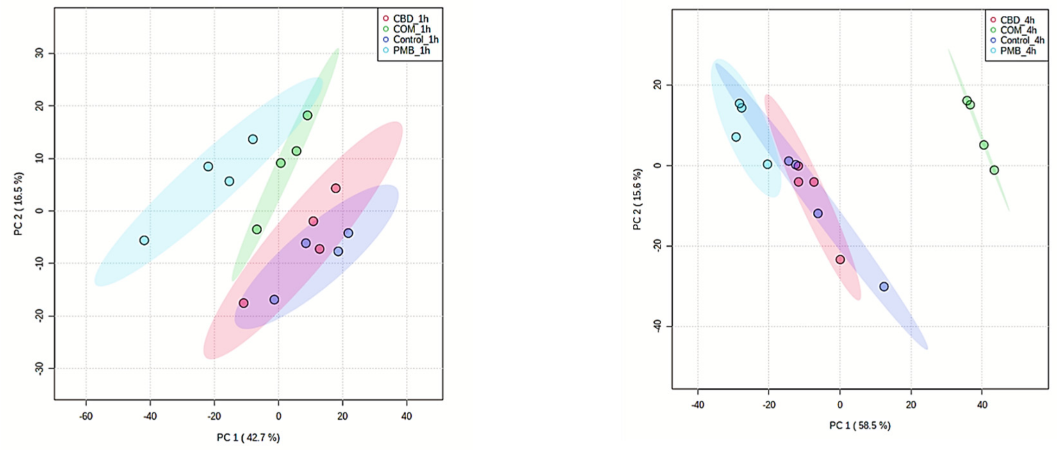

(B)

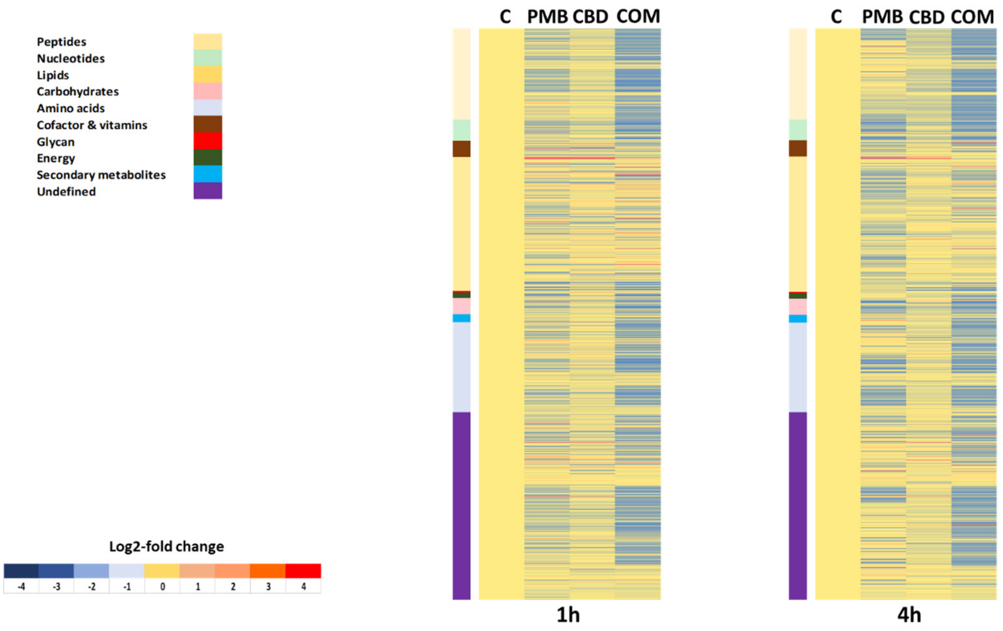

Figure S2. (A) PCA plots for metabolite levels from *A. baumannii* ATCC 19606 samples treated with polymyxin B (PMB), cannabidiol (CBD), and their combination (COM) at 1 and 4h. Each data set represents a total of 8 samples of 4 biological replicates of each condition. Purple = control; Cyan = polymyxin B (PMB); Red= cannabidiol (CBD); Green= combination. **(B)** Heatmap profiles of *A. baumannii* ATCC 19606 with hierarchical clustering of all identified metabolites after treatment with polymyxin B (PMB), cannabidiol (CBD), and their combination (COM) at 1 and 4h.

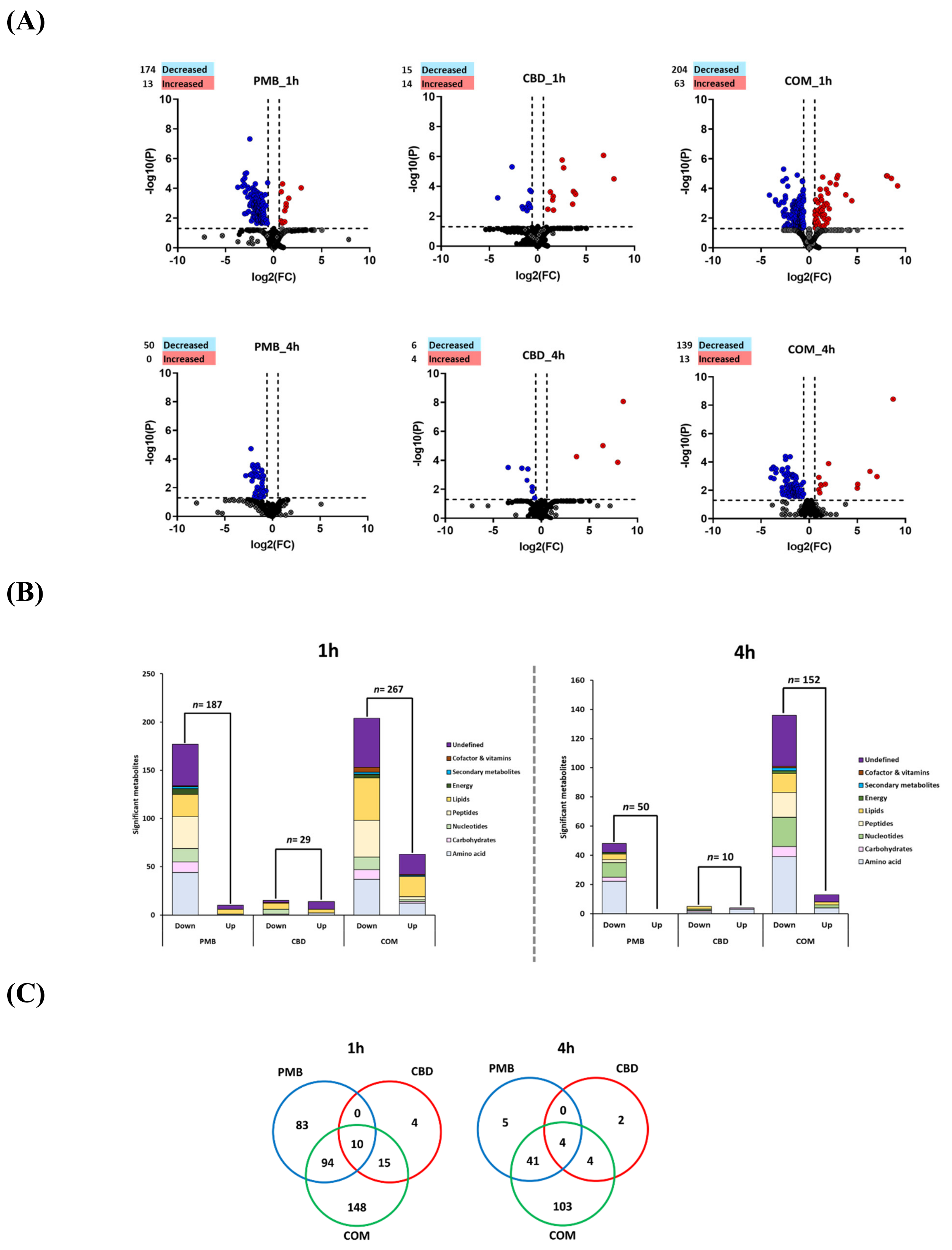

Figure S3. (A) Volcano plots show the total number of significant metabolites after antibiotic monotherapy and combination treatment of *A. baumannii* ATCC 19606 at 1 and 4h. **(B)** The summary of significantly changed metabolites of *A. baumannii* ATCC 19606 from different categories following PMB and CBD monotherapies and their combination treatment at 1 and 4h. Changes (≥ 0.58553-log2-fold, $p \leq 0.05$). **(C)** Venn diagrams showing the number of metabolites significantly affected by each treatment for *A. baumannii* ATCC 19606 at 1 and 4. Significant metabolites were selected with (≥ 0.59-log2-fold, $p \leq 0.05$).

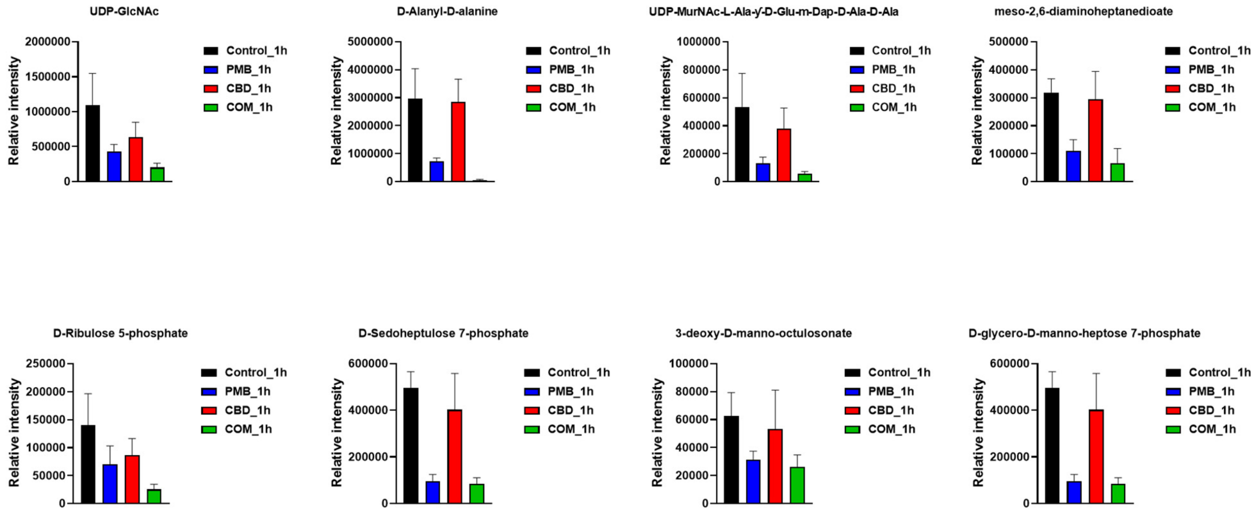

Figure S4. The bar charts of significantly impacted metabolites of amino-sugar and nucleotide-sugar metabolism, PPP and downstream peptidoglycan and LPS biosynthesis for *A. baumannii* ATCC 19606 treated with polymyxin B (PMB) or cannabidiol (CBD) monotherapy and the combination (COM) after 1h exposure (≥ 1.0-log_2-fold, $p \leq 0.05$).

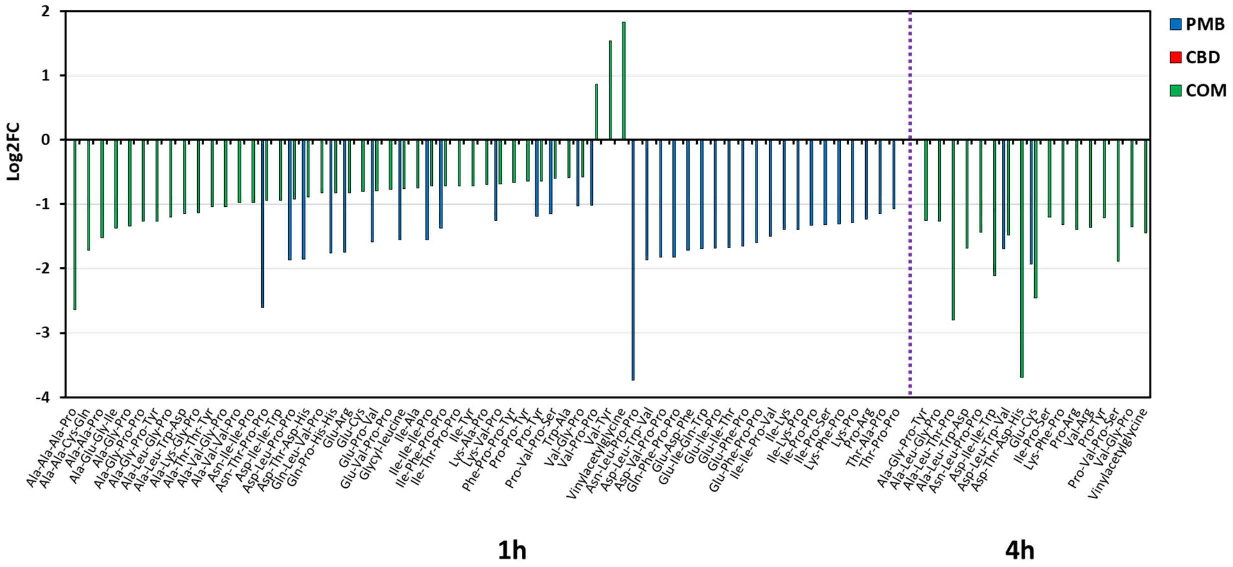

Figure S5. The fold change charts of significantly impacted metabolites of peptides metabolism for *A. baumannii* ATCC 19606 treated with polymyxin B (PMB) or cannabidiol (CBD) monotherapy and the combination (COM) after 1 and 4h exposure (≥ 1.0-log_2-fold, $p \leq 0.05$).
